# Noncirrhotic hyperammonemia: A factor behind dementia to alter mental status

**DOI:** 10.1002/ccr3.2436

**Published:** 2019-09-27

**Authors:** Christopher Leo, Yun Wang, Alexander Mold, Junik Quintana, Hong Shi, Mahdi Abdullah, Dariush Alaie, Richard Petrillo

**Affiliations:** ^1^ Department of Medicine Montefiore Mount Vernon Hospital Mount Vernon NY

**Keywords:** dementia, elderly, hyperammonemia, mental status

## Abstract

Healthcare givers were recommended to check serum ammonia level for elderly patients with acute‐on‐chronic alteration of mental status. Early initiation of antihyperammonemia therapy may benefit improvement of alteration of mental status. Baseline mental status becomes necessary for diagnose the acute alteration of mental status and monitor the therapeutic process.

## Graphical abstract

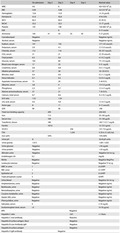

## 1 CASE PRESENTATION

Reported was a case regarding noncirrhotic hyperammonemia with an unknown etiology in an elderly patient. Empirical treatment with lactulose for hyperammonemia was initiated and turned out to be effective for improving mental status. Now in details, a 68‐year‐old Caucasian male was admitted to our hospital presenting with altered mental status, aggressive behavior, agitation, and suicide ideation for 2 days. The patient stated “I feel like I cannot control myself ….. I might hurt myself or you.” He even tried to attack medical staff during the patient encounter. He was having some difficulty orienting to place and time. When asked why he was in the hospital, the patient said “they do not let me use that computer. They played game, but don't let me in."

The patient was severely anxious and unable to stop walking. He went to the restroom twelve times within 1 hour, and had no bowel movement or urination. There was no history of head trauma, fall, gastrointestinal bleeds, or bacteremia. Patient denied loss of consciousness, headache, dyspnea, chest pain, muscle weakness, muscle pain, numbness, or tingling. His medical history included dementia, schizophrenia and chronic constipation, depression, and anxiety. His past surgical history was noncontributory. His home medication included olanzapine (10 mg oral, twice daily), simethicone (80 mg as needed), Senna lax (8.6 mg 2 tabs at bedtime), docusate sodium (100 mg 2 tab at bedtime), milk of magnesia (400 mg/5 mL oral suspension give 30 mL by oral, as needed if no BM by 8 pm on day 2). The patient had no known drug or food allergies. He lived in a skilled nursing home. He denied smoking, drinking alcohol, or use of any recreational drugs.

When admitted, patient's vital signs were as follows: oral temperature 97.6 degree Fahrenheit (F), heart rate 70 beats per minute, blood pressure 131/76 mm Hg, and mean 94 mm Hg. Respiratory rate was 17 breaths per minute, and oxygen saturation was 98% on room air. His weight was 179.89 pounds. His body mass index was 27.3 kg/m^2^ and body surface area (BSA) 1.9 m^2^. He was oriented only to himself, but not to time and place. He was able to follow simple commands.

Physical examination revealed a confused patient in no acute distress. He had no change of sensation. His muscle strength was 4/5 on upper and lower extremities. His cranial nerves were intact. He had no slurred speech. No conjunctiva injection, pale sclera, limited range of motion, joint swelling, or tenderness was noticed. His breath sounds were diminished but without wheezing, crackles, or rales bilaterally. Normal S1 and S2 heart sounds were heard, with no murmur, gallops, or clicks. His abdomen was soft and nontender. No distention, rigidity, rebound, or guarding was found.

The initial laboratory results of the patient are listed in Table 1. His serum ammonia was 104 µmol/L on admission. Liver function test was in normal ranges: aspartate transaminase 15 IU/L (International Units Per Liter), alanine aminotransferase 16 IU/L, total bilirubin 0.4 mg/dL, and direct bilirubin 0.1 mg/dL. His urine toxicology test was negative for cannabinoid, amphetamine, barbiturate, benzodiazepine, cocaine metabolite, methadone, opiate, phencyclidine, and salicylate. Serum acetaminophen level was lower than 5 µm/mL. Serum alcohol level was nondetectable. Hemoglobin was 13.8 gm/dL. The computed tomography scan of head without contrast showed mild periventricular white matter ischemic changes but no intracranial hemorrhage. The ultrasound of abdomen revealed no significant abnormality, except increased echogenicity of liver consistent with fatty infiltration.

**Table 1:** Laboratory results over the hospitalization

	On admission	Day 2	Day 3	Day 4	Day 5	Normal value
WBC	4.6		6			4.8‐10.8 10^3^ µL
RBC	5.04		5.08			4.4‐5.9 10^6^ µL
Hemoglobin	13.8		13.9			14‐18 gm/dL
Hematocrit	41.6		42.8			41%‐53%
MCV	82.5		84.3			83‐98 fL
MCHC	33.2		32.5			33‐37 gm/dL
Platelet	145		148			130‐400 10^3^ µL
ESR			8			0‐15 mm/hr
Ammonia	104	41	43	35	30	9‐33 µmol/L
Cannabompod	Negative					Negative ng/mL
Alcohol, serum	Negative					Negative ng/mL
Sodium, serum	141		142			137‐145 mmol/L
Potassium, serum	3.9		4.1			3.5‐5.0 mmol/L
Chloride, serum	112		110			98‐107 mmol/L
CO2, serum	21		21			22‐30 mmol/L
Total protein	6.3		6.6			6.3‐8.2 mg/dL
Glucose, serum	103		84			75‐110 mg/dL
Blood urea nitrogen, serum	17		23			9‐21 mg/dL
Creatinine, serum	0.8		0.8			0.8‐1.5 mg/dL
Alkaline phosphatase	57		58			38‐126 IU/L
Bilirubin, total	0.4		0.6			0.2‐1.3 mg/dL
Direct bilirubin	0.1		0.2			0.0‐0.4 mg/dL
Aspartate transaminase, serum	15		20			5‐40 IU/L
Albumin, serum	3.8		4			3.9‐5.0 gm/dL
Phosphorus	2.3		3.7			2.5‐4.5 mg/dL
Alanine aminotrasferase, serum	57		21			7‐56 IU/L
Calcium, total serum	8.7		8.6			8.4‐10.2 mg/dL
A/G ratio	1.52		1.54			
Uric acid, serum	4.4		4.8			3.5‐8.5 mg/dL
Anion gap	8		11			8‐12 mmol/L
GFR	90		90			>90
Total iron binding capacity		209				250‐425
Iron		113				50‐180 µg/dL
Serum Iron		109				35‐140 µg/dL
Transferrin, Serum		180				180.7‐313.7 mg/dL
Ferritin		107.6				14‐235 ng/mL
Vit B12				256		143‐716 pg/mL
TSH				1.234		0.38‐6.15 mIU/mL
Iron sat%		54%				15%‐60%
Urine pH	8					4.6‐8 pH units
Urine gravity	1.015					1.001‐1.035
Urine glucose	Negative					<50 mg/dL
Urine protein	Negative					<30 mg/dL
Bilirubin urine	Negative					Negative Sm to Lg
Urobilinogen UA	0.2					mg/dL
Nitrite	Negative					Negative Neg/Pos
Leukocyte esterase	Negative					Negative Tr to Lg
WBC in urine	1					0‐2/HPF
RBC urine	1					0‐1/HPF
Epithelial cell	2					0‐3/HPF
Triple phosphate crystal	5					0/HPF
Urine blood	Negative					Negative Sm to Lg
Amphentamine, urine	Negative					Negative ng/mL
Barbiturate, urine	Negative					Negative ng/mL
Benzodiazepine, urine	Negative					Negative ng/mL
Cocaine metabolite, urine	Negative					Negative ng/mL
Opiate 300, urine	Negative					Negative ng/mL
Phencyclidine, urine	Negative					Negative ng/mL
Salicylate, serum	0					2‐30 mg/dL
Acetaminophen level, serum	<5					10‐30 µg/mL
RPR				Negative		
Hepatitis C ratio				2.81		<1 Ratio
Hepatitis C viral antibody				Reactive		
Hepatitis B surface antigen, Neut				Negative		
Hepatitis B core Antibody IgM				Negative		
Hepatitis B surface antigen				Negative		
Hepatitis A IgM antibody			Negative			

Note

Cannabompod: cutoff = 50; Amphentamine, urine: cutoff = 1000 ng/mL; Barbiturate, urine: cutoff = 200 ng/mL; Benzodiazepine, urine: cutoff = 200 ng/mL; Cocaine metabolite, urine: cutoff = 300 ng/mL; Opiate 300, urine: cutoff = 300 ng/mL; Phencyclidine, urine: cutoff = 25 ng/mL.

On the management with lactulose (30 mL oral, twice daily), his home medications and 1:1 observation, the patients experienced gradual improvement over the following 3 days, but still had intermittent agitation and aggressive behavior. His body temperature was in the range of 97‐98 degree F. He never had oxygen desaturation during hospitalization. On the fourth day of hospitalization, the patient became more calm, alert, and cooperative and able to communicate with doctor and nurse effectively. His serum ammonia dropped down to 35 µmol/L. The 1:1 observation was thus canceled. Patient was still confused, but the patient's primary care doctor confirmed that patient has come back to his baseline mental status. After kept at his baseline for additional 1 day, patient was discharged and went back to a nursing home. He followed up with his primary care doctor within 1 week after discharge with stable status.

## 2 DISCUSSION

Altered mental status (AMS) is a medical term describing any change in mental functioning involving consciousness or cognition or both. Clinically, patients may present with a spectrum from inattentiveness, confusion, altered behavior, agitation, to the status of coma. Elderly patients are more susceptible to experience altered mental status, mostly because of them having multiple vulnerability factors, such as old age, dementia, comorbidities, and functional dependence.^1^ These patients’ vulnerabilities may contribute to altered mental status with some precipitating factors, including infection, electrolyte abnormalities, organ failure, intoxication, thyroid dysfunction, dehydration, central never damage, trauma, and medications.^2^

Ammonia is able to cross the blood‐brain barrier and causes altered mental status or encephalopathy when accumulated in blood that was defined as hyperammonemia, an elevation of serum ammonia level. The etiologies of primary hyperammonemia include defect of enzymes in urea cycle, organic acidemias, fatty acid oxidation defects, and dibasic amino acid transport defects, which are commonly seen in children.^2^ In adults, the secondary hyperammonemia is more common, especially in the presence of hepatic disorders. It may also happen with normal hepatic function when mitochondrial pathways are interrupted by Reye's syndrome 3 or medications, such as valproic acid,^4^, ^5^, ^6^, ^7^, ^8^ carbamazepine,^9^ salicylate,^10^ topiramate,^5^ or cytotoxic agents.^11^, ^12^, ^13^ Additionally, renal tubular acidosis,^14^ urinary tract dilatation 5 or urinary tract infection,^15^, ^16^ pregnancy, and hypoglycin 2 may contribute to nonhepatic hyperammonemia.

Nonhepatic hyperammonemia is a diagnosis of exclusion for a cause of encephalopathy. In this case, our patient had no anemia. His liver enzymes, blood urea nitrogen (BUN), creatinine were in normal range. Glomerula filtration rate (GFR) was over 90. He did not take any medications that may contribute to hyperammonemia based on the previous reports. His hepatitis C virus (HCV) ratio was 2.8, but no HCV RNA (Ribonucleic acid) was detected in blood, meaning a latent HCV infection. His constipation was resolved with multiple stool softeners. No nausea, vomiting, or urination problem was noticed. These implied that the hyperammonemia in this patient was nonhepatic. More important, patient's alteration of mental status occurred with hyperammonemia, and the mental status was significantly improved when the serum ammonia level dropped back to normal range. The is suggested that the nonhepatic hyperammonemia might be the cause of the acute onset of altered mental status in this patient.

This patient has history of dementia and schizophrenia. These are two medical problems significantly predisposing to altered mental status. Therefore, identifying the factors causing altered mental status in the patients with dementia or schizophrenia or both can be challenging. In dementia, the alteration of mental status is mostly irreversible and progressing slowly (months to years). Patients with dementia may present with impairment of cognition involving language, memory, learning, social cognition, executive functions, or attention.^17^ It must weaken independence in daily activities, but not occur exclusively during delirium or other mental disorders, such as schizophrenia. Schizophrenia shares some similar clinical features with dementia, such as impairment attention, memory, or executive functions. But schizophrenia is well characterized by hallucinations or delusions, disorganized speech. A period of at least 6 months is required for diagnosing this disease. In this case, the patient presented with confusion, but may answer questions logically. He clearly knew that he was unable to control his aggressive behavior. His agitation and aggressive behavior occurred in the past 2 days, compared with his baseline mental status. The short period of these symptoms suggested dementia and schizophrenia were less likely to be the cause of AMS. It was supported by the subsequent psychiatry clearance to the patient.

A well‐known acute status of altered mental status is delirium. Delirium is a reversible change of mental status and may occur and progress rapidly (hours to days). Patients with delirium may present with inattention, agitation, aggressive behavior or thinking, and disorganized speech, which were the patient's symptoms in this case. It has been reported that valproic acid may induce delirium due to hyperammonemia.^4^, ^5^, ^6^, ^7^, ^8^ In our case, the patient was given Haldol to control delirium and lactulose to reduce serum ammonia. The patient's mental status was significantly improved when serum ammonia level decreased back to normal range, even when Haldol was used less frequently. These suggested that (a) hyperammonemia might be the cause of acutely altered mental status in this case; (b) empirical treatment for hyperammonemia may benefit improvement of altered mental status, especially when patients have hyperammonemia with unidentified etiologies.

Taken together, we recommended (a) testing serum ammonia level should be a part of a standard workup for elderly patients with acute alteration of mental status; (b) once hyperammonemia is determined, correcting the ammonia level may benefit improvement of mental status; (c) patient's baseline mental status is an important factor that helps to diagnose acute AMS in patient with dementia or schizophrenia, and monitor treatment effects of AMS.

## AUTHOR CONTRIBUTIONS

CL: wrote the manuscript. CL, YW, HS, and MA: involved in a primary care team of this case. AM and JK: collected the data and the references. DA and RP: involved as resident mentors for the primary care team and provided advices for publication.
